# Antioxidant Activity of Telmisartan–Cu(II) Nanoparticles Connected 2-Pyrimidinamine and Their Evaluation of Cytotoxicity Activities

**DOI:** 10.1155/2020/8872479

**Published:** 2020-11-18

**Authors:** Radhakrishnan Surendrakumar, Akbar Idhayadhulla, Saud Alarifi, Nazeer Anis Ahamed, Chidambaram Sathish Kumar

**Affiliations:** ^1^Research Department of Chemistry, Nehru Memorial College (Affiliated to Bharathidasan University), Puthanampatti, Tiruchirappalli District, 621007 Tamil Nadu, South India, India; ^2^Department of Zoology, College of Sciences, King Saud University (KSU), P.O. Box 2455, Riyadh 11451, Saudi Arabia; ^3^Department of Botany & Microbiology, College of Sciences, King Saud University (KSU), Riyadh, Saudi Arabia

## Abstract

Copper nanoparticles (Cu-Nps) are one of the promising materials for the advancement of nanoscience and technology. In this work, we synthesized telmisartan copper nanoparticles and 2-pyrimidinamines via Biginelli reaction using telmisartan copper nanoparticles (Cu-Nps) as a reusable catalyst. The synthesis of 2-pyrimidinamine derivatives (1a-c) was achieved in water and under solvent-free condition (Green chemistry approach). Synthesis of 2-pyrimidinamine with telmisartan copper nanoparticle (Cu-Nps–Pyr) unexpected product was also isolated from synthesis of 2-pyrimidinamine preparation. Antioxidant and cytotoxic activities were carried out both in 2-pyrimidinamine (1a-1c) and 2-pyrimidinamine with telmisartan copper nanoparticles (Cu-Nps–Pyr). The synthesized 2-pyrimidinamine derivatives (1a-c) were characterized from FT-IR, ^1^H and ^13^C NMR spectroscopy, mass and elemental analyses. The synthesized telmisartan copper nanoparticles (Cu-Nps) were characterized from UV spectroscopy, XRD, SEM, EDX, AFM (atomic force microscopy), profile, waviness, and roughness analyses. Antioxidant activity was screened based on ABTS^·+^ radical scavenging and linoleic acid peroxidation performance. Cu-Nps–Pyr-1b showed substantial antioxidant (97.2%) activity against ABTS^·+^ assay and 91.2% activity against AAPH assays compared with Trolox. Cytotoxicity was evaluated using HepG2, HeLa, and MCF-7 cell lines, the Cu-Nps–Pyr-1a is high in toxicities (GI_50_ = 0.01 *μ*m) against the HeLa cancel cell line compared with doxorubicin. The developed copper NPs with 2-pyrimidinamine (Cu-Nps–Pyr) could provide promising advances as antioxidant activities; this nanocomposition could be considered an anticancer treatment in future investigations.

## 1. Introduction

**Figure 1:**
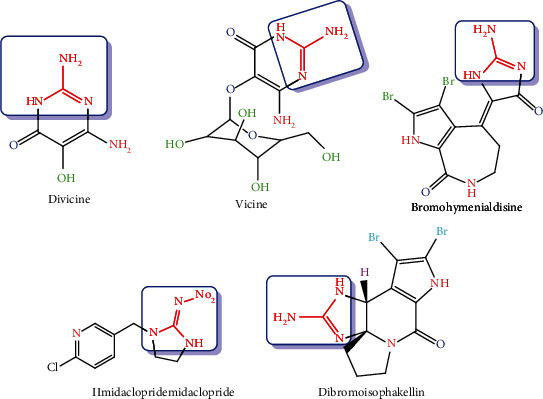
Biologically active natural pyrimidinamine compounds.

Pyrimidinamine has great importance as it is widely spread in living organisms and also a broad spectrum of biological activities such as antitumor properties [1, 2], herbicidal [3], fungicidal [4], antidepressant [5], and also, amino pyrimidine was an indicator of antitumor [6], antibacterial [7], and antiviral [8] activities. The biological importance of natural and synthetic 2-pyrimidinamine core derivatives are shown in Figure 1, such as alkaloidal (divicine) properties found in fava beans, the aglycone of vicine [9], the cytotoxic activity of alkaloid marine sponge (bromohymenialdisine) [10], and antifeedant of alkaloid (dibromoisophakellin) [11].

**Figure 2:**
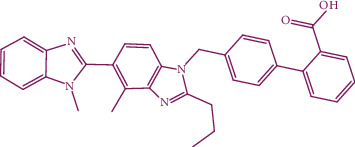
Structure of telmisartan.

Telmisartan is used in the management of hypertension, which acts as an angiotensin II receptor blocker (ARB). The structure of telmisartan is shown in Figure 2 and their orally active direct-acting AII, the AT1receptor antagonist potential of pharmacotherapy of hypertension [12].

Scavenging ROS antioxidants of acetyl cysteine additionally L-carnitine are observed to cure the inflammatory diseases [13, 14]. However, it does not performed well against ROS-related diseases due to poor bioavailability and various limitations of clinical screening [15, 16]. Recently, studies reported that copper-based nanoparticles improved the ROS-related diseases and higher scavenging ability of this treatment [17]. Copper (Cu) is one of the best elements in recommendation for human usage, which play an important role in tyrosinase and Cu–ZnSOD enzyme [18, 19]. Cu enzyme activity is basically a response to many bioactivities such as connective tissue biosynthesis, cellular respiration, neurotransmitter production, iron homeostasis, peptide biogenesis, pigment formation, and antioxidant defense [20, 21]. Particularly, Cu-NPs has outstanding catalytic activity to scavenge H_2_O_2_ and O_2_ [22] but cannot eliminate OH· simultaneously [23] and also can promote electron transfer reactions to inactivate H_2_O_2_ or OH· [24]. The catalytic behavior of Cu compounds in organic reaction motivated us to focus our attention on the application of our research. As a result, we assume that the capacity of Cu-Nps acts as catalytic as well as antioxidant activities simultaneously. In addition, the stability of Cu-NPs can be largely enhanced, which is also developing the overall ROS scavenging ability [25, 26]. Based on the above observation, we designed to synthesize, high active 2-pyrimidinamine connected with Cu nanoparticles and evaluated the antioxidant of ABTS^·+^, linoleic acid peroxidation, and cytotoxic activities.

## 2. Material and Methods

### 2.1. General Materials and Characterization Techniques

#### 2.1.1. Chemistry

Telmisartan-mediated copper nanoparticle was analyzed from UV, FT-IR, SEM, X-ray with EDX, and AFM methods. FT-IR is recorded via Shimadzu 8201pc (4000-400 cm^−1^). The ^1^H and ^13^C NMR were recorded via JEOL-300 MHz. An elemental analyzer model (Varian EL III) was used for analysis of elemental presences. Thin layer chromatography was used for purity checking.

### 2.2. Synthesis of Telmisartan-Mediated Copper Nanoparticles

The mixture of CuCl_2_·2H_2_O (0.5 mmol, 5 mL) and telmisartan (1 mmol, 10 mL) was added in ethanol; then, the reaction mixture was allowed 1 M NaOH with stirring and maintained pH = 7. The reaction mixture converted blue precipitate followed by adding of water (15 mL). The precipitate was obtained, then filtered all precipitate and purified with suitable techniques.

### 2.3. General Method for Preparation of 2-Pyrimidinamine Derivatives (1a-1c) via Biginelli Reaction

The compound urea (0.01 mol, 0.6 g), Cu catalyst (0.01 mol, 1.7 g), ethyl acetoacetate (0.01 mol, 1.3 mL), benzaldehyde (0.01 mol, 1 mL), and substituted amine (0.05 mol, 1.6 mL) are mixed well together in a mortar and two drops of the con. HCl were added. The precipitate was dissolved in ethanol. The undissolved catalyst was filtered and dried in vacuum and used for the next cycle. The final product was obtained from the evaporation of ethanol from the filtrate and unexpected product also obtained. The final compound was confirmed by TLC. The pure product was obtained from the using suitable recrystallized method. The above procedure was followed for synthesis of the remaining compounds 1b and 1c.

### 2.4. Recyclability of Cu-Np Catalyst

Investigation of recovery the catalyst and usage via washing and dried with ethanol by under vacuum condition are shown.

#### 2.4.1. Ethyl 2-hydrazono-6-methyl-4-phenyl-1,2,3,4-tetrahydropyrimidine-5-carboxylate (1a)

The composition of 1a is as follows: black solid; mw: 274.32; mp 136°C; IR (cm^−1^): 3246.11 (NH), 3119.59 (Ph-CHstr), 3033.92 (Ar-H), 2597.00 (C=N), 1713.57 (OCOEt); ^1^H NMR (300 MHz, DMSO- d_6_): *δ* 9.20 (2H, s, NH), 8.84 (2H, s, NH_2_), 7.33(2H, dd, *J* = 7.33Hz, *J* = 7.37 Hz Ph), 7.28 (1H, d, *J* = 6.21 Hz, phenyl), 7.23(2H, dd, *J* = 7.31 Hz, *J* = 7.35 Hz Ph), 4.20 (2H, m, -CH_2_) 4.16 (1H, d, *J* = 6.21 Hz, -CH), 2.26 (3H, s, -CH_3_), 1.29 (3H, s, -CH_3_); ^13^C NMR(300 MHz, DMSO-d_6_): 167.2 (1C, C=O), 160.3(1C, -C-CH_3_), 154.0 (1C, C=N), 143.3, 128.5, 126.9, 126.7 (6C, Ar ring), 104.2 (1C, -C-CO-), 61.7(1C, -C-CH_3_), 45.1 (1C, -C-NH), 18.4 (1C, -CH_3_), 14.1 (1C, -CH_3_); elemental analysis: Calcd. for C_14_H_18_N_4_O_2_: elemental analysis: C, 61.30; H, 6.61; N, 20.42%; found: C, 61.27; H, 6.62; N, 20.45%.

#### 2.4.2. Ethyl 6-methyl-4-phenyl-2-(2-phenylhydrazono)-1,2,3,4-tetrahydropyrimidine-5-carboxylate (1b)

The composition of 1b is as follows: grey solid; mw: 350.41; mp:184°C; IR (cm^−1^): 3239.20 (NH), 3128.82 (Ph-CHstr), 2596.43 (C=N), 1715.02 (OCOEt); ^1^H NMR (300 MHz, DMSO- d_6_): *δ* 11.12 (1H, s, NH), 9.34 (2H, s, NH), 7.35 (2H, dd, *J* = 7.33 Hz, *J* = 7.37 Hz Ph), 7.33 (2H, dd, *J* = 7.33 Hz, *J* = 7.37 Hz Ph), 7.29 (1H, d, *J* = 6.21 Hz, Phenyl), 7.23 (2H, dd, *J* = 7.31 Hz, *J* = 7.35 Hz Ph), 7.20 (2H, dd, *J* = 7.31 Hz, *J* = 7.35 Hz Ph), 6.81 (1H, d, *J* = 6.21 Hz, phenyl), 4.20 (2H, m, - CH_2_) 4.12 (1H, d, *J* = 6.21 Hz, -CH), 2.26 (3H, s, -CH_3_), 1.29 (3H, s, -CH_3_); ^13^C NMR (300 MHz, DMSO-d_6_): 167.2 (1C, C=O), 160.3(1C, -C-CH_3_), 154.0 (1C, C=N), 143.3, 128.5, 126.9, 126.7(6C, Ar ring), 143.0, 113.9, 129.5, 122.4 (6C, Ar ring), 104.2 (1C, -C-CO-), 61.7 (1C, -C-CH_3_), 45.1(1C, -C-NH), 18.4 (1C, -CH_3_) 14.1 (1C, -CH_3_); elemental analysis: Calcd. for C_20_H_22_N_4_O_2_: C, 68.55; H, 6.33; N, 15.99 %; found: C, 68.52; H, 6.37; N, 15.98%.

#### 2.4.3. Ethyl 2-(carbamothioylimino)-6-methyl-4-phenyl-1,2,3,4-tetrahydropyrimidine-5-carboxylate (1c)

The composition of 1c is as follows: white solid; mw: 318.39; mp:236°C; IR (cm^−1^): 3239.02 (NH), 3128.82 (Ph-CHstr), 2596.43 (C=N), 1715.02 (OCOEt), 1094.82 (C=S); ^1^H NMR (300 MHz, DMSO- d_6_): *δ* 9.26 (2H, s, NH), 8.84 (2H, s, NH_2_), 7.33 (2H, dd, *J* = 7.33 Hz, *J* = 7.37 Hz Ph), 7.25 (1H, d, *J* = 6.21 Hz, phenyl), 7.23 (2H, dd, *J* = 7.31 Hz, *J* = 7.35 Hz Ph), 4.20 (2H, m, - CH_2_), 4.10 (1H, d, *J* = 6.21 Hz, -CH), 2.26 (3H, s, -CH_3_), 1.29 (3H, s, -CH_3_); ^13^C NMR(300 MHz, DMSO-d_6_): 190.0 (1C, C=S), 167.2 (1C, C=O), 160.3(1C, -C-CH_3_), 163.0 (1C, C=N), 143.3, 128.5, 126.9, 126.7 (6C, Ar ring), 104.2 (1C, -C-CO-), 61.7 (1C, -C-CH_3_), 45.1 (1C, -C-NH), 18.4 (1C, -CH_3_), 14.1 (1C, -CH_3_); elemental analysis: Calcd. for C_15_H_18_N_4_O_2_S: C, 56.58; H, 5.70; N, 17.60%; found: C, 56.60; H, 5.71; N, 17.57%.

### 2.5. Antioxidant–(ABTS^·+^) Scavenging Activity

ABTS^+^ (2,2′-azino-bis(3-ethylbenzothiazoline-6-sulfonicacid)), the antioxidant radical scavenging activity, was checked with all compounds via spectrophotometric analysis according to the method descripted in Surendra Kumar et al. [27]. The ABTS^·+^ radical scavenging is established and calculated the ability of antioxidants via characteristic absorption at 734 nm for long-lived ABTS radical cation, which indicated that blue/green chromophore. The results are shown in Table 1. The tested reaction mixture was prepared by 7 mM aqueous ABTS with 2.45 mM potassium per sulfate at 12 h maintained in the dark room with formal temperature. Prior to the experiment, a range of absorbance 0.700 ± 0.025 at 734 nm was obtained by the ABTS^·**+**^ solution was diluted with phosphate buffer (0.1 M, pH 7.4) and added with 1 mL of the diluted 1.5 mL ethanolic solutions of 1a-c, Cu-Nps, and Cu-Nps-Pyr (1a-1c) with different concentrations (100 *μ*g/mL). After 30 min, the percentage of inhibition was calculated at 734 nm. Ethanol is used as a blank sample, following the formula used for analysis of the percentage of inhibition.

(1) $$\begin{array}{c} \text{ABTS}+\text{scavenging}\text{effect}\left(\%\right)=\left[\frac{\left(A_{\text{c}}–A_{\text{s}}\right)}{A_{\text{c}}}\right]\times 100\%, \end{array}$$

where *A*_c_ = initial ABTS^·**+**^ absorbance and *A*_s_ =1a–c, Cu-Nps, and Cu-Nps-Pyr (1a-1c) absorbance.

### 2.6. Inhibition of AAPH Assay Free Radical Analysis

This linoleic acid peroxidation assay was achieved and followed the methods of Surendra Kumar et al. [27]. All values were carried out in triplicate, with the consistent data, provided in Table 1. The oxidation of linoleic acid production was analyzed by conjugated diene hydroperoxide in an aqueous distribution was observed at 234 nm, [28] with AAPH (2,2′-azobis(2-amidinopropane) dihydrochloride) used as a free radical initiator. For UV cuvette solution preparation, 1 mL aliquot of a 16 mM linoleic acid was added to 1 mL of 0.05 M phosphate buffer(pH 7.4), thermostatic at 37°C and addition of 1 mL of the 40 mM AAPH solution. Oxidation reaction was monitored in occurrence of 1 mL of the tested compounds 1a-c, Cu-Nps, and Cu-Nps-Pyr (1a-1c). In the absence of antioxidants, the lipid oxidation was monitored in DMSO mixed compounds. The rate of oxidation was monitored at 37°C and absorption recorded at 234 nm due to the formation of a conjugated diene hydroperoxide.

The percentage of linoleic acid oxidation inhibition was calculated as follows:

(2) $$\begin{array}{c} \%\text{Inhibition}=\left[\frac{\left(1–\text{rate}\text{of}\text{absorbance}\text{with}\text{test}\text{compound}\right)}{\text{rate}\text{of}\text{absorbance}\text{with}\text{solvent}\text{control}}\right]\times 100\%. \end{array}$$

### 2.7. Cytotoxic Activity

The synthesized compounds 1a-c, Cu-Nps, and Cu-Nps -Pyr (1a-1c) were screened for cytotoxic activity, and we followed the methods of Surendra Kumar et al. [27, 29]. The 100 *μ*M (MTT anticancer assay) concentration treated with test compounds for 48 h was used by three cell lines (HepG2, HeLa, and MCF-7), and standard of doxorubicin was selected in this screening.

The results were analyzed between the growth percentage of treated cells and untreated control cells. Sample preparation, 0.1 mL of the test solution (6.25–100 *μ*g in 1% DMSO), and 0.1 mL aliquot of the cell suspension (5 × 10^6^ cells/100 *μ*L) were added and kept in an incubator (5% CO_2_) at 37°C for 72 h. The blank sample checked only the cell suspension, and the control wells contained 1% DMSO and the cell suspension. After 72 h, 20 *μ*L of MTT was added, and the plates were kept in the CO_2_ incubator for 2 h, followed by the addition of propanol (100 *μ*L). The absorption data (562 nm) were obtained by 27-well plates on an ELISA reader; the test sample was prepared by aluminum foil covered with subsequently agitated in a rotary shaker for 10–20 min.

### 2.8. Statistical Analysis

Antioxidant activities results were calculated through 3 independent evaluations, and Microsoft Excel was used to analysis the standard deviations (SD) of each compound.

## 3. Result and Discussion

The one-pot multicomponent 2-pyrimidinamine was synthesized with in high yields. The most important factor was only excellent yield, which was a performance of suitable catalysis with aromatic aldehyde performance including hydroxyl groups reacted efficiently.

**Scheme 1:**
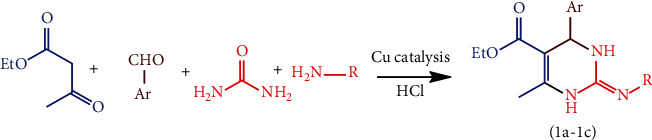
Synthetic route of 2-pyrimidinamine derivatives.

A series of compounds were prepared via optimizing the reaction conditions, for instance of reaction involved among benzaldehyde, ethyl acetoacetate, substituted amine, and urea using Cu-Nps as a catalyst in a suitable solvent (Scheme 1). The amount of Cu-Nps was changed subsequently from 0 mol% to 5 mol% under solvent-free conditions, as a result of the optimized mole percentage and the 88% yield performance obtained at 1 mol% concentration. Therefore, the results directed that 1 mol% of Cu-Nps is enough for the synthesis of the 2-pyrimidinamine compound. The synthesized Cu-Nps were characterized by UV, FT-IR spectroscopy, SEM, XRD, EDX, AFM (atomic force microscopy), profile, waviness, and roughness analyses.

### 3.1. FT-IR Studies

**Figure 3:**
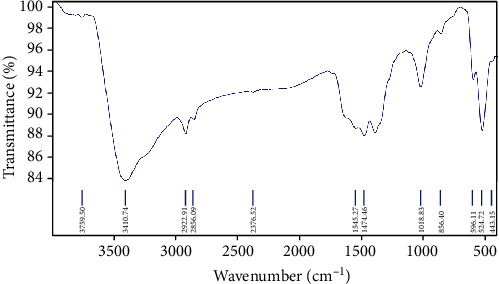
FT-IR spectrum for telmisartan-mediated copper nanoparticle.

The main characteristic experimental vibrations of Cu-Nps are obtained from FT-IR shown in Figure 3. The literature data of IR values were referred to the analysis of the vibrational frequency presence in the compounds. For spectral values of O−H and C−H stretching, the vibration frequency range was obtained at 4000−2000 cm^−1^. The O−H stretching in water molecules was assigned at 3432 cm^−1^ for Cu-Nps. The mode of the antisymmetric and the symmetric carboxylate groups was observed at 1593 cm^−1^ (s, FT-IR) and 1391 cm^−1^(vs. FT-IR), respectively. Biphenyl group was found in the IR range between 1600 and 1400 cm^−1^, respectively. The COOH bends and C-N (Bz) stretching modes was observed at 1235 cm^−1,^ respectively.

### 3.2. UV-Visible Studies

**Figure 4:**
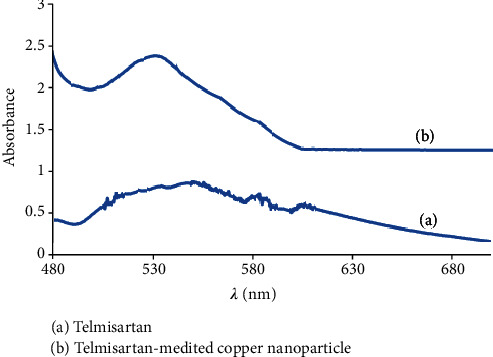
UV-vis spectra of Cu-Nps.

Spectrophotometric analysis was carried out for the Cu-Np catalyst. 6.25 mM of telmisartan was added with different copper concentrations with the addition of 1 M NaOH (pH 7) for every tested compound. The UV-visible spectra are shown in Figure 4. The Cu-Nps showed absorption bands located at 530 nm (sh).

### 3.3. SEM and EDX Analyses

**Figure 5:**
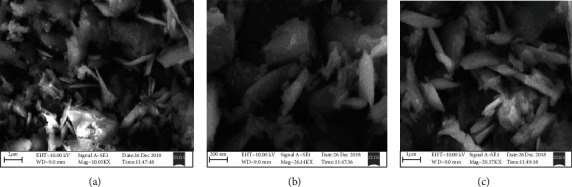
Telmisartan Cu-Nps of SEM images.

**Figure 6:**
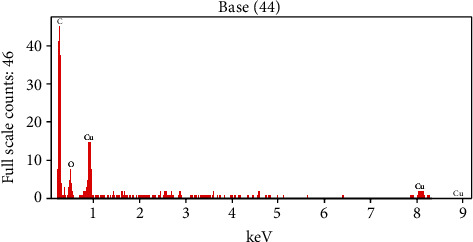
Telmisartan-copper nanoparticle of ED.

Figure 5 illustrates SEM images of Cu-Nps obtained in water (a) and in formamide mixture (b). The telmisartan-mediated Cu-Nps in the formamide mixture shows cubic in nature with size in the range of 1 *μ*m (c).  Figure 6 illustrates the EDX of Cu-Nps that exhibited the elements (Cu, C, and O) in the nanoparticle, thereby confirming the formation of the nanoparticle.  Table 2 shows the EDX analysis of percentage of elements present in the Cu-Np catalyst.

### 3.4. Powder X-Ray Diffraction Studies

**Figure 7:**
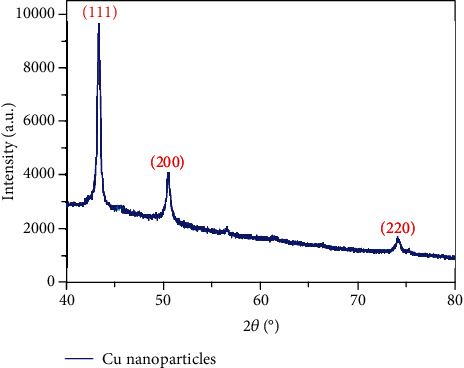
XRD studies of telmisartan-mediated copper nanoparticle.

The structural analysis and phase crystallinity of the synthesized Cu-Nps were examined via the powder X-ray diffraction method.  Figure 7 signifies a Cu nanoparticle diffraction pattern at a 2*θ* value of 43.20°, 50.42°, and 74.15° respect to (111), (200), and (220) planes individually; it conformed the formation of copper cubic lattice. JCPDS No. 040836 indicates the good agreement and comparison with the standard pattern for pure face centered cubic phase of copper nanoparticles. There are no impurity peaks observed. The strong peaks indicate the highly crystalline nature of the formed nanoparticles. From the observed main diffracted peak, the average crystalline size can be calculated using the Scherer equation.

(3) $$\begin{array}{c} D_{\left(\text{hkl}\right)}=\frac{k\lambda }{\beta \cos\theta }, \end{array}$$

where *D*_(hkl)_ is the average crystalline size, *k* is shape constant (0.89), *λ* is the incident X-ray (CuK*α* source, *λ* = 0.15405 nm), *β* is the full-width half-maximum, and *θ* is the incident angle of X-ray. The average crystallite size of the synthesized copper nanoparticles was 25.41 nm.

### 3.5. Atomic Force Microscope Studies

**Figure 8:**
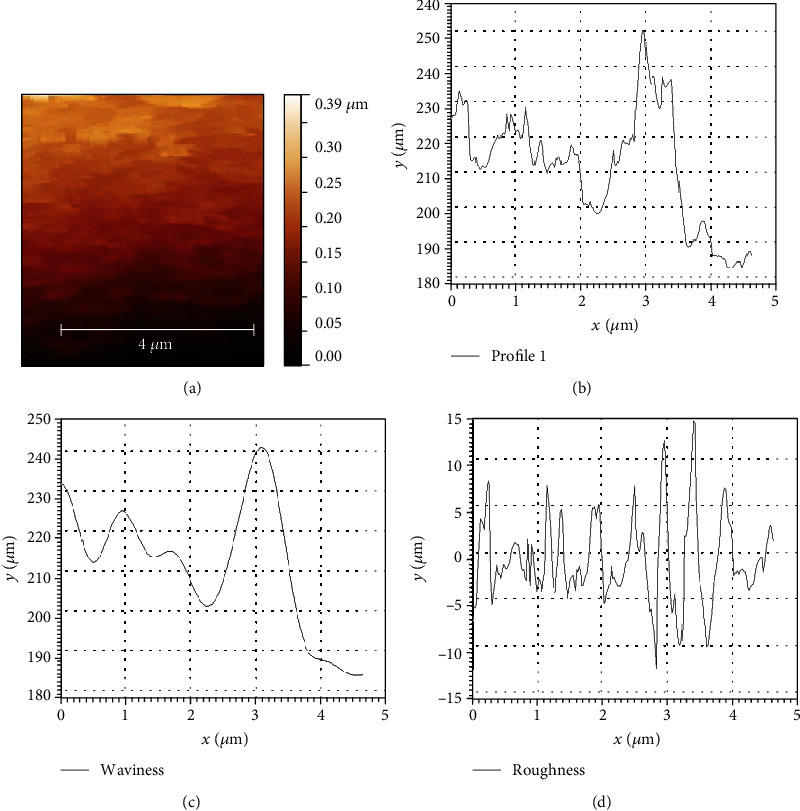
(a) AFM image of Cu-Np catalyst. (b) The profile of Cu in telmisartan-mediated copper nanaoparicles. (c) The waviness of Cu-Nps. (d) The roughness of Cu-Nps.

Figure 8 shows AFM images of Cu-Nsp. AFM image indicates the filtering procedure at cut-off frequency values at 4 *μ*m and Cu-Nsp profile particle size; performance is presented in Figure 8(b), which obtained a value of 253 nm for the unfiltered profile. Figures 8(c) and 8(d) indicated the Cu-Np waviness and roughness, which were 234 nm for waviness and 14 nm for the roughness profile. Therefore, such values indicate that the unfiltered roughness parameter of shape and binding dominate Cu nanoparticles.

### 3.6. TGA/DTA Studies

**Figure 9:**
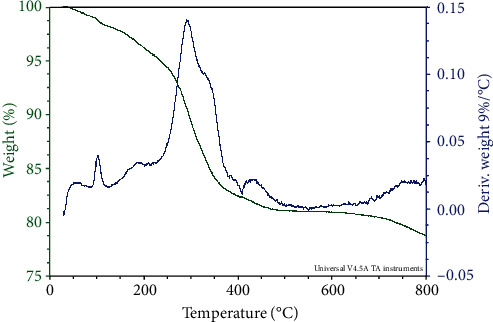
TG/DTA curve of Cu-Np precursor.

The compound was displayed by total weight loss in three stages. In the first stage, the weight loss occurred in the temperature range about 40-220°C representing the vapor liberation in the sample. The weight loss befell in the second stage at the temperature range between 220 and 450°C consistent to the liberation of vapor from the inner apertures of Cu. The weight loss in the third stage happens between the temperature ranges of 450-800°C display indulgences of carbonaceous material of the sample. Organic material presence in the sample, was observed at exothermic peak of 290°C. The TGA/DTA profile for the Cu-Np precursor was shown in Figure 9.

### 3.7. Particle Size Analyzer Studies

**Figure 10:**
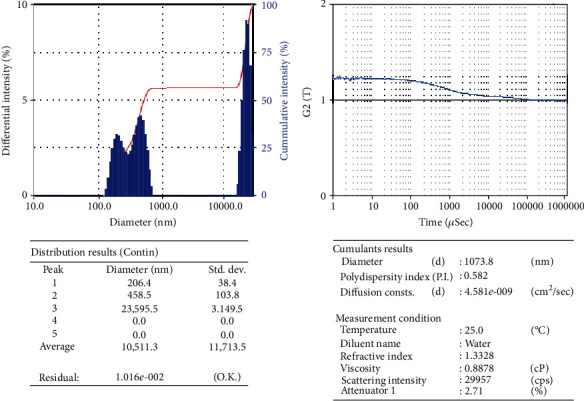
Particle size analysis of Cu-Nps.

For a particle size analyzer, the first peak indicates 206.4 nm diameter and standard deviation 38.4 nm, which indicates 206.4 nm average nanoparticle presence in the target nanocatalysis.  Figure 10 shows different intensity vs. diameter of the first peak of Cu-Nps.

### 3.8. Catalyst Recovery Studies

**Figure 11:**
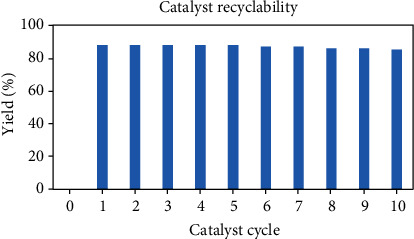
Recyclability of telmisartan copper nanoparticle catalyst.

Figure 11 indicated the recovery of the catalyst with salvaged for at least 10 run times and slight loss in catalytic activity. The lessening of activity could be detected with the regenerated catalyst on salvaging due to surface area of the catalyst during the reaction or partial loss of basic sites/regeneration. The values are shown in Table 3.

**Scheme 2:**
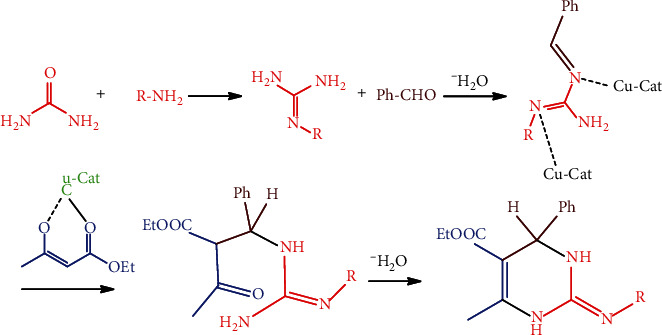
Catalysis performance with mechanism of compound preparation **(**1a-1c).

The application of the catalyst was inspected via optimizing the reaction conditions. A number of aromatic aldehydes were selected to go through the condensation reaction with Cu-Nps (1 mol %) catalytic at room temperature in solvent-free settings, the yield reported in Table 3. The Cu-catalyzed performance and reaction mechanism are shown in Scheme 2.

The intermediate of acyl imine formed between urea reacts with primary amine; for the next step, acyl imine reacted with aldehyde to give the guanidine core, which is actuated by copper catalysis. Step 3 was addition reaction between *β*-dicarbonyl with 1-benzylidene-2-R-guanidine, then cyclized to afford the 2-aminopyrimidine derivatives. In conclusion, the simple and efficient synthesis of 2-aminopyrimidine uses Cu-Nps under solvent-free conditions. We developed the methodology and environmentally safe synthesis of the 2-aminopyrimidine derivative with good yields.

Compounds 1a-1c were synthesized by condensation method. The compounds 1a-1c physicochemical data are represented in Table 4 and its catalyst performance is shown in Table 5. The compounds were confirmed via FT-IR, ^1^H and ^3^C NMR spectra, and elemental analyses. The IR absorption bands of compound 1a show the frequency range between 2584 and 2596 and 3239 and 3246 cm^−1^ corresponding to the C=N and NH groups, respectively.

For compound 1a, the ^1^H NMR spectral value obtained a sharp singlet at *δ* 9.20 for NH proton and a singlet at *δ* 4.16 for -CH- proton, ^13^C NMR spectral values at *δ* 167.2 and *δ* 154.0 ppm corresponding to C=O and C=N carbons, respectively. The IR absorption bands show that compound 1b frequency ranges between 3239 and 3246 and 2584 and 2596 cm^−1^ corresponding to the NH and C=N groups, respectively. For compound 1b, ^1^H NMR spectral value obtained a sharp singlet at *δ* 9.34 for NH proton and a singlet at *δ* 4.12 for -CH- proton, ^13^C NMR spectra of compound peaks at *δ* 167.0 and *δ*154.8 ppm corresponding to C=O and C=N carbons, respectively.

For compound 1c, IR spectra show absorption bands in 2584–2596 and 3239-3246 cm^−1^ corresponding to the C=N and NH groups, respectively. For compound 1c, ^1^H NMR spectral value obtained a sharp singlet at *δ* 9.26 for NH proton and a singlet at *δ* 4.10 for -CH- proton; ^13^C NMR spectral value obtained the peaks at *δ* 163.0, 167.2, and 190.0 ppm corresponding to C=N, C=O, and C=S carbons, respectively.

**Scheme 3:**
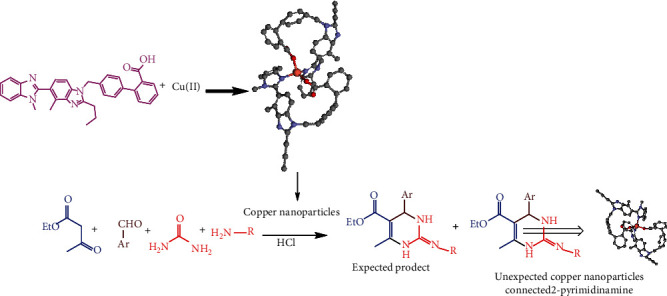
Mechanism of expected product and unreacted unexpected copper nanoparticles connected 2-pyrimidine compounds.

Scheme 3 indicates that the mechanism of expected product and unexpected copper nanoparticles connected 2-pyrimidine compounds; this mechanism clearly indicated the necessity for evaluation of the biological activity in 1a–c, Cu-Nps, and unexpected copper nanoparticles connected 2-pyrimidine Cu-Nps-Pyr (1a-1c) compounds.

### 3.9. ABTS^·+^ Radical Scavenging Activity

The antioxidant activity was carried out based on a decolorization technique using the ABTS radical assay, with the generation of the stable blue/green ABTS^·+^ radical formation. Among the synthesized compounds, Cu-Nps-Pyr-1b showed capable ABTS^·+^ scavenging activities of 97.2%, compared with Trolox standard (Table 1).

**Scheme 4:**
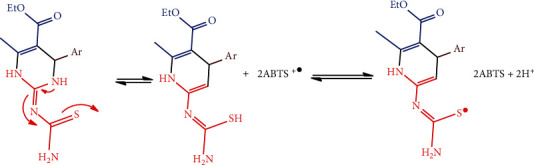
ABTS^·+^ radical scavenging—mechanism of inhibition.

This study, containing both 2-pyrimidinamine and Cu-Nps, shows the capability to scavenge ABTS^·+^ assays compared with standard; the mechanism is represented in Scheme 4.

### 3.10. Cu-Nps–Pyr-1c-Linoleic Acid Peroxidation-Inhibition

**Scheme 5:**
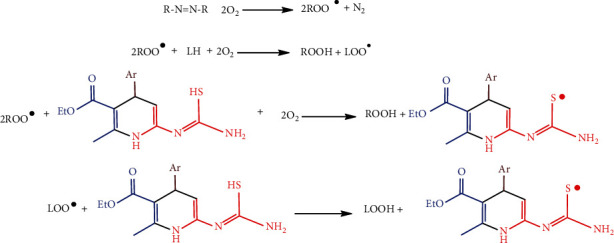
Lipid peroxidation—mechanism of inhibition.

The Cu-Nsp with 2-pyrimidinamine combination was highly responsive to oxidative changes and inhibition in linoleic acid peroxidation screening. The hydrophilic AAPH initiator was used to measure the conjugated diene hydroperoxide formation at 234 nm by spectrophotometric analysis. Indeed, AAPH produced the free radicals at 37°C, which was an act of spontaneous decomposition. Here, R–N=N–R indicates the radical initiator, and LOO^·^ denotes a linoleic peroxy radical, and LH stands for linoleic acid. The radicals R^·^ was produced via immediately reacting with oxygen and causing the oxidation of lipids. The mechanism of reaction is represented in Scheme 5.

The Cu-Nsp with 2-pyrimidinamine derivatives was performed by hydrogen donor antioxidants (AOH), steadying and postponing the free radicals of oxidation lipids. In *in vitro* studies of free radical fabrication, an azo compound was causing free radicals by impulsive thermal decomposition. Alkylperoxy free radicals were produced via the water-soluble AAPH used as a hygienic and governable source thermally. AAPH assay was achieved to enhance the antioxidant activity of Cu-Nsp with 2-pyrimidinamine; the compound 1c and Cu-Nps-Pyr-1c are awarding activities of 75.6 and 91.2%, respectively, at a concentration of 100 *μ*g/mL.

### 3.11. Cytotoxic Activity

The results of each compound were reported in Table 6. The results show the contacts of LC_50_, TGI, and GI_50_ values. The compound 1a (GI_50_ = 0.69*μ*M) was high, while Cu-Nps-Pyr-1a (GI_50_ = 0.01 *μ*m) showed a significant activity against the HeLa cancer cell line. The compound 1b (GI_50_ = 02.1 *μ*M) and Cu-Nps-Pyr-1b (GI_50_ = 0.01 *μ*M) showed rightful activity against the HepG2 cell line compared with standard doxorubicin. All compounds displayed a moderate activity against all the cell line, whereas when compound with Cu nanoparticles, it is becoming highly responsive in cytotoxic scanning.

## 4. Conclusion

In conclusion, for copper nanoparticles mediated by the telmisartan drug, the Cu-Nsp was characterized by UV and FT-IR spectroscopy, scanning electron microscopy, X-Ray, EDX, AFM (atomic force microscopy), profile, waviness, roughness analysis, TGA studies and particle size analyzer. Synthesized Cu-Nps were used as catalysis for preparation of pyrimidinamine via the Biginelli multicomponent cyclocondensation reaction. The Cu-Nsp was a significantly active catalyst and gave excellent yields, and also recycling of the catalyst is very easy in this process. Bioactivity of 2-pyrimidinamine was a significant activity against performance of ABTS^·+^ and linoleic acid peroxidation, while Cu-Nsp was moderately active in antioxidant screening; therefore, designed 2-pyrimidinamine with Cu-Nsp gave a noble result when compared with commercial drugs. Particularly, Cu-Nps-Pyr-1b was a potential activity (>90%) against ABTS^·+^ and AAPH assays, as compared with the Trolox standard. In cytotoxicity activity, the compound Cu-Nps-Pyr-1a was perceived for (GI_50_ = 0.01 *μ*m) against the HeLa cell line compared with doxorubicin. It is noteworthy that Cu-Nps with 2-pyrimidinamine and their derivatives are medicinally important as anticancer treatment, and future investigation of *in vivo* screening and the mechanism of action is currently undergoing study.

## Figures and Tables

**Table 1:** Antioxidant activities of pyrimidinamine derivatives 1a-c, Cu-N, and Cu-N-Pyr.

Compounds	Percentage of activity (%)^a^	
	ABTS^·*+*^	AAPH
1a	54.3 ± 0.07	64.2 ± 0.61
1b	61.0 ± 0.12	72.0 ± 0.11
1c	80.8 ± 0.12	71.6 ± 0.24
Cu-Nps	28.3 ± 0.10	37.9 ± 0.32
Cu-Nps –Pyr-1a	72.1 ± 0.23	78.3 ± 0.61
Cu-Nps –Pyr-1b	86.3 ± 0.13	80.3 ± 0.42
Cu-Nps –Pyr-1c	97.2 ± 0.20	91.2 ± 0.28
Trolox	85.2 ± 0.09	62.3 ± 0.28

^a^Values are the meansofthreereplicates ± SD.

**Table 2:** EDX analysis of percentage of elements present in Cu-Nps.

Element line	Weight (%)	Weight % error	Atom (%)
C K	74.54	±3.03	82.93
O K	18.75	±2.92	15.66
Cu K	6.72	±3.54	1.41
Cu L	—	—	—
Total	100.00		100.00

**Table 3:** Catalyst recyclability.

Entry	Catalyst use	Yield (%)
1	1^st^	88
2	2^nd^	88
3	3^rd^	88
4	4^th^	88
5	5^th^	88
6	6^th^	87
7	7^th^	87
8	8^th^	86
9	9^th^	86
10	10^th^	85

**Table 4:** Optimization of reaction condition.

S. no.	R	Ar	Reaction condition	Catalyst mole (%)	Yield (%)	Final product structure
1a	-NH_2_	Ph	10 min, RT	1.0	88	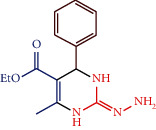
1b	-NH-Ph	Ph	10 min, RT	1.0	88	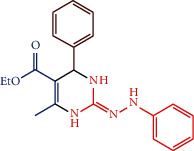
1c	-CS-NH_2_	Ph	10 min, RT	1.0	88	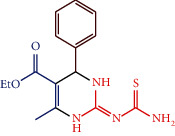

**Table 5:** Optimization of catalyst of Cu-Nps.

S. No.	Catalyst	Mole (%)	Yield (%)	Yield (%) of Cu-N-Pyr
1	Telmisartan	1	56	0
2	Cu-Nps	5	94	0.6
3	Cu-Nps	4	92	0.8
4	Cu-Nps	3	90	10
5	Cu-Nps	2	89	11
6	Cu-Nps	1	88	12

**Table 6:** Cytotoxic activities of pyrimidinamine derivatives 1a-c, Cu-N, and Cu-N-Pyr.

Compounds	HepG2			MCF-7			HeLa		
	GI_50_ (*μ*M)	TGI (*μ*M)	LC_50_ (*μ*M)	GI_50_ (*μ*M)	TGI (*μ*M)	LC_50_ (*μ*M)	GI_50_ (*μ*M)	TGI (*μ*M)	LC_50_ (*μ*M)
1a	03.2	09.3	32.1	0.06	0.62	12.0	0.69	12.3	25.6
1b	02.1	06.1	24.1	12.2	24.3	42.2	0.09	20.3	41.2
1c	06.3	14.3	44.1	10.2	20.3	41.3	12.3	24.6	46.2
Cu-Nps	12.3	24.2	45.3	18.5	36.2	69.6	21.0	46.3	84.0
Cu-Nps-Pyr-1a	0.02	0.21	0.59	0.04	0.28	0.81	0.01	0.19	0.42
Cu-Nps-Pyr-1b	0.01	06.1	24.1	12.2	24.3	42.2	0.12	0.33	0.92
Cu-Nps-Pyr-1c	0.05	14.3	44.1	0.02	0.13	0.53	0.05	0.16	0.36
Doxorubicin	0.01	0.13	0.58	0.02	0.21	0.74	0.05	0.41	0.88

## Data Availability

The data used to support the findings of this study are available from the corresponding author upon request.
